# Associations between serum levels of brain-derived neurotrophic factor, corticotropin releasing hormone and mental distress in vitiligo patients

**DOI:** 10.1038/s41598-022-11028-8

**Published:** 2022-05-04

**Authors:** Assiya Kussainova, Laura Kassym, Almira Akhmetova, Eugenia Dvoryankova, Natalya Glushkova, Zaituna Khismetova, Saltanat Adilgozhina, Raikhan Tuleutayeva, Alida Kaskabayeva, Meruyert Massabayeva, Laura Pak, Yuliya Semenova

**Affiliations:** 1 Department of Dermatovenerology and Cosmetology, Semey Medical University, Semey, Kazakhstan; 2 School of Medicine, Nazarbayev University, 5/1 Kerey&Zhanibek Khandar Street, Nur-Sultan, 010000 Republic of Kazakhstan; 3 Centre of Theoretical Problems of Physico-Chemical Pharmacology, Russian Academy of Sciences, Moscow, Russia; 4 Department of Epidemiology, Biostatistics and Evidence based medicine, Al-Farabi Kazakh National University, Almaty, Kazakhstan; 5 Department of Public Health, Semey Medical University, Semey, Kazakhstan; 6 Department of Family Medicine, Semey Medical University, Semey, Kazakhstan; 7 Department of Pharmacology, Semey Medical University, Semey, Kazakhstan; 8 Department of Faculty Therapy, Semey Medical University, Semey, Kazakhstan; 9 Scientific Research Laboratory, Semey Medical University, Semey, Kazakhstan; 10 Department of Clinical Oncology and Nuclear Medicine, Semey Medical University, Semey, Kazakhstan; 11 Department of Neurology, Ophthalmology, Otorhinolaryngology, Semey Medical University, Semey, Kazakhstan

**Keywords:** Psychology, Vitiligo

## Abstract

Vitiligo is clinically characterized by the appearance of non-symptomatic depigmented macules, but the disorder is highly correlated with a wide range of psychiatric disorders and psychological problems. The aim of our study was to investigate serum brain-derived neurotrophic factor (BDNF) and corticotropin releasing hormone (CRH) levels in vitiligo patients and healthy controls in relation to the observed symptoms of depression and anxiety disorders. This study comprised 96 vitiligo patients and 96 healthy controls who filled out the Patient Health Questionnaire-9 (PHQ-9) and Generalized Anxiety Disorder-7 (GAD-7) scales. Serum levels of BDNF and CRH were measured using enzyme-linked immunosorbent assay (ELISA) technique. There was a significant increase of depression and anxiety scores in vitiligo patients as compared with healthy controls (P < 0.05). The serum levels of BDNF were significantly lower in vitiligo patients than in healthy individuals (Z = 4.002; P < 0.001), while the serum levels of CRH were markedly higher in cases than those in controls (Z = 3.764; P < 0.001). The significant positive correlations between serum CRH levels and GAD-7, PHQ-9 scores were observed. However, the aforementioned psychometric scales did not correlate significantly with serum BDNF level. Vitiligo is associated with the depression and is closely linked with lower BDNF levels.

Vitiligo is a common autoimmune disease which affects pigment producing melanocytes, resulting in the appearance of visible whitish patches on skin and mucosal membranes^1^. The prevalence of vitiligo varies from 0.5% to 2% worldwide^2^. The origin of vitiligo is unknown though the pathogenesis is associated with the intrinsic abnormalities of melanocytes, oxidative stress, and sympathetic neurogenic imbalances^3^. Vitiligo is clinically characterized by the appearance of non-symptomatic depigmented macules, but the disorder is highly correlated with a wide range of psychiatric disorders and psychological problems^4^. Because of emotional and social burden associated with vitiligo, numerous studies investigating relationship between psychiatric disturbances and manifestation, course, and severity of the disease have been conducted. Individuals with vitiligo suffer from depressive disorders, stigmatization, emotional and behavioral impairment, sleep disturbance, and decreased quality of life^5–8^. In addition, a meta-analysis reported that patients with vitiligo have the same risk of anxiety as compared to subjects suffering from eczema, psoriasis, and acne^9^. Certainly, the pathogenetic relationship between vitiligo and mental impairments needs to be considered in detail. A better understanding of mechanisms of neuroendocrine and inflammatory disturbances in vitiligo might be achieved by investigation of some crucial signaling substances.

Corticotropin releasing hormone (CRH) is the important hypothalamic factor which regulates the pituitary secretion of adrenocorticotropic hormone (ACTH). As the crucial part of the hypothalamo-pituitary-adrenal (HPA) axis, CRH is the driver of the stress response. The role of CRH is well described in pathogenesis of some autoimmune diseases, neurological and mental abnormalities^10^. Some studies suggest that CRH is increased in depression and anxiety disorders. For example, Cao S et al. (2020) found that the serum levels of CRH was significantly higher in women with postpartum depression (PPD) than in the non-PPD group. The authors offered that the serum levels of CRH and serotonin may serve as a potential early marker for identifying women at high risk for PPD^11^. To date, there are practically no studies devoted to determine the serum levels of CRH in vitiligo patients. Some authors described the cutaneous expression of CRH and its’ receptor, which is detected by performing a skin biopsy. The authors found a significant increase in the expression of CRH and corticotropin releasing hormone receptor 1 (CRH-R1) in both damaged and intact skin of vitiligo patients that were exposed to stress^12^. Besides, the in vitro studies and clinical evidence of direct and indirect pro-inflammatory action of CRH were reported for vitiligo. A recent research found that CRH may also contribute greatly to skin homeostasis. The CRH activates regional mast cells and its receptor (CRH-R1) is expressed on melanocytes. In its turn, the damage of melanocytes causes the hypopigmentation process^13^. In addition, the cutaneous CRH increases the production and extrication of pro-opiomelanocortin (POMC), which is involved in melanogenesis^14^.

Brain-derived neurotrophic factor (BDNF) is another important agent which may act as potential regulator of neuropsychiatric processes. It plays vital role in different stages of neurons’ life cycle, such as growth, survival, differentiation, and repair^15^. BDNF has been established already as the possible biomarker for the onset and progression of major depressive disorder (MDD), bipolar disorder, schizophrenia, Alzheimer disease, Parkinson’s disease, and epilepsy^16^. The above-mentioned spectrum of neuropsychiatric abnormalities was conceivably associated with the decreased BDNF levels. In addition to that, some chronic somatic conditions are also linked with the reduced level of BDNF. For instance, Tschorn et al. (2020) reported the lower BDNF levels in patients with chronic heart failure^17^. Similarly, the serum levels of BDNF were investigated in chronic skin disorders. Such, Sjahrir et al. (2019) showed that a low level of serum BDNF may increase severity of both depression and psoriasis vulgaris^18^. Besides, recent research reported the decreased level of BDNF in patients with vitiligo^19^. Thus, the aim of our study was to investigate serum BDNF and CRH levels in vitiligo patients and healthy controls in relation to the observed symptoms of depression and anxiety disorders.

## Methods

### Subjects and study proceedings

This study was carried out during a 6-month period from October 2020 to March 2021. The participants were adult patients who were referred to Dermatology department of General Hospital #2 of Semey City, Kazakhstan. The study was approved by the local Ethics Committee of Semey Medical University (Protocol #2, from 18 October 2019), and the research was conducted in compliance with principles of the Declaration of Helsinki and the Guideline for Good Clinical Practice. All participants provided written informed consent. Totally, 192 individuals aged 16–79 years were selected for the study based on inclusion/exclusion criteria. The participants were divided into two groups: 96 patients with a confirmed diagnosis of vitiligo and 96 healthy controls. Exclusion criteria for patients or healthy individuals included: subjects who were younger than 16 years of age, who were currently treated for a psychiatric disorder, who had another dermatologic disease or severe concomitant pathology (liver dysfunction, alcoholism, drug addiction, cancer), and who did not want to participate in the study.

### Enrollment and assessment criteria

All patients were subjected to complete history taking regarding their age, sex, ethnicity, educational level, duration and activity of the disease, history of vitiligo in parents or grandparents, previous treatment, general and dermatological examination in daylight and using a Wood lamp to determine Fitzpatrick's skin photo type. The type of vitiligo and the percentage of affected body area were determined by the Vitiligo Extent Score (VES) associated with mucosal lesions, leukotrichia, Koebner's phenomenon and halo-nevi^20^.

### Laboratory tests

Five milliliters of venous blood samples were withdrawn from each participant on an empty stomach in the morning (before 10.00 AM) using serum separator tubes and left for 30 min at room temperature to allow clotting and then centrifuged for 15 min at 3000 rpm. All serum samples were collected and then stored for less than 6 months at −20 °C. A double-antibody sandwich enzyme-linked immunosorbent assay (ELISA) technique was used to determine BDNF serum levels with the help of a commercial kit (Serial #: 9F1F9F01E4, Cloud-Clone Corp., USA). Measurement of CRH was also performed by ELISA kit (Serial #: 09A102086A, Cloud-Clone Corp., USA) according to the same technique. All manufacturer instructions were strictly followed.

### Evaluation of depression and anxiety

Depression was evaluated on the basis of the Patient Health Questionnaire-9 (PHQ-9), which is an internationally recognized screening tool. The PHQ-9 consists of nine questions that are based on the DSM-IV criteria for a MDD. The questionnaire explores the symptoms experienced by patients during the two immediately preceding weeks. The scores for each PHQ-9 item range from 0 (not at all), to 3 (nearly every day). Total scores of 5, 10, 15, and 20 indicate the presence of mild, moderate, moderately severe and severe disorder, respectively. MDD should be considered in patients who endorse ≥ 5 of the 9 symptoms as present “more than half the days” (the 9^th^ item counts if endorsed “several days”) and one of the first two symptoms (depressed mood or loss of interest) is endorsed^21^. Likewise, the anxiety was evaluated with the help of the Generalized Anxiety Disorder-7 (GAD-7) scale, which is also a well-recognized international screening tool. The GAD-7 scores range from 0 to 27, with 5, 10 and 15 representing mild, moderate and severe levels of anxiety symptoms^22^. Previously, both tools were successfully utilized in the Kazakhstani population^23^.

### Statistical analysis

Qualitative data presented as absolute numbers and their percentages. The test of difference in groups of nominal data was Chi square test. All numerical variables were tested by the Kolmogorov–Smirnov test for normality of distribution. Since the observed data did not follow the normal distribution, the quantitative data were expressed as median and 25th-75th percentiles. For difference testing in groups of quantitative variables, we used a Mann Whitney U-test. To find any possible correlation between the variables, we used the Spearmen's correlation test. Receiver operating curve and logistic regression procedure were used to analyze a predictive significance of laboratory markers (BDNF and CRH). The associations between vitiligo and BDNF/CRH levels were assessed in relation to a patient gender and anxiety/depression scores. The critical value of statistical significance was at a probability of α less than 5%. All statistical procedures were performed in SPSS 20.

## Results

Table 1 contains the data related to the demographic and clinical characteristics of the vitiligo patients and healthy controls. In this study, 96 patients with vitiligo (59.4% females, 97.9% Kazakhs) and 96 healthy participants (55.2% females, 89.6% Kazakhs) were compared. There was no difference between the groups in terms of age, gender, ethnicity, and education level (all P > 0.05). Nearly half of the participants were married, and more than the half of them had both parents. The study cohort was mostly represented by the second and the third Fitzpatrick skin types (48.4% and 31.8%, respectively).

**Table 1:** Baseline characteristics in subjects with vitiligo and healthy control groups.

Variables		Group				Test of difference	
		Healthy controls		Cases			
		N	%	N	%	Statistical	p-value
Age (years), mean ± standard error*	39 ± 2	38 ± 2	0.602	0.548			
Gender	Females	57	59.4	53	55.2	0.341**	0.559
	Males	39	40.6	43	44.8		
Ethnicity	Kazakh	94	97.9	86	89.6	5.810**	0.055
	Russian	2	2.1	9	9.4		
	Other	0	0.0	1	1.0		
Education level	HS or less	8	8.3	6	6.3	2.252**	0.324
	College or less	32	33.3	42	43.8		
	HE or less	56	58.3	48	50.0		
Marital status	Single	35	36.5	28	29.2	1.482**	0.686
	Married	47	49.0	55	57.3		
	Divorced	7	7.3	6	6.3		
	Widow	7	7.3	7	7.3		
Having parents	Have both parents	58	60.4	52	54.2	1.025**	0.599
	There is only one parent	22	22.9	23	24.0		
	No parents	16	16.7	21	21.9		
Fitzpatrick skin type	1	10	10.4	9	9.4	1.527**	0.676
	2	47	49.0	46	47.9		
	3	32	33.3	29	30.2		
	4	7	7.3	12	12.5		

*Test of difference was t-test.

**Test of difference was Chi-square test.

HS—Higher School; HE—Higher Education.

The patients with vitiligo demonstrated significantly higher anxiety and depression scores (all P < 0.05). The serum level of BDNF was significantly lower in vitiligo patients as compared with the healthy controls (2.60 (1.88–3.01) vs. 3.23 (2.52–4.33); P < 0.001). Median serum CRH level was 6.39 (2.46–8.31) in vitiligo patients and 3.24 (2.18–3.85) in healthy subjects (P < 0.001) (Table 2).

**Table 2:** Associations between anxiety and depression scores and serum BDNF, CRH levels in vitiligo patients and healthy controls.

Variables	Group		Test of difference*
	Healthy controls	Cases	p-value
	Median (Q1–Q3)		
**Anxiety and depression scales**			
GAD-7	1.00 (0.00–3.00)	4.00 (2.00–7.00)	< 0.001
PHQ-9	0.00 (0.00–2.00)	2.00 (1.00–3.00)	0.013
**Neurotransmitters**			
BDNF	3.23 (2.52–4.33)	2.60 (1.88–3.01)	< 0.001
CRH	3.24 (2.18–3.85)	6.39 (2.46–8.31)	< 0.001

*—test of difference was Mann Whitney U-test.

GAD-7—Generalized Anxiety Disorder 7-item Scale.

PHQ-9—The Patient Health Questionnaire 9-item depression module.

BDNF – Brain-derived neurotrophic factor.

CRH – Corticotropin releasing hormone.

Female vitiligo patients had significant higher GAD-7 scores as compared with male patients: 3.0 (2.0–6.0) vs.5.0 (3.0–7.0) (P = 0.032) (Table 3).

**Table 3:** Characteristics of vitiligo patients stratified by gender.

Variables	Gender		Test of difference*
	Females	Males	p-value
	Median (Q1–Q3)		
**Anxiety and depression scales**			
GAD-7	5.00 (3.00–7.00)	3.00 (2.00–6.00)	0.032
PHQ-9	2.00 (1.00–4.00)	1.00 (0.00–3.00)	0.234
**Neurotransmitters**			
BDNF	2.58 (1.83–2.97)	2.63 (2.14–3.24)	0.575
CRH	6.56 (2.87–8.38)	5.92 (2.34–8.02)	0.313

*—test of difference was Mann Whitney U-test.

GAD-7—Generalized Anxiety Disorder 7-item Scale.

PHQ-9—The Patient Health Questionnaire 9-item depression module.

BDNF – Brain-derived neurotrophic factor.

CRH – Corticotropin releasing hormone.

Anxiety level correlated positively with PHQ-9 score (r = 0.296; P = 0.003). We detected statistically significant positive weak correlations between serum CRH level and GAD-7 scores (r = 0.214; P = 0.036) (Table 4).

**Table 4:** Correlations between anxiety, depression scores, serum BDNF and CRH levels.

	GAD-7	PHQ-9	BDNF	CRH
GAD-7	*r* = 1	*r* = 0.296	*r* = - 0.010	*r* = 0.214
		*P* = 0.003	*P* = 0.921	*P* = 0.036
PHQ-9	*r* = 0.296	*r* = 1	*r* = 0.044	*r* = 0.152
	*P* = 0.003		*P* = 0.671	*P* = 0.138
BDNF	*r* =−0.010	*r* = 0.044	*r* = 1	*r* = - 0.042
	*P* = 0.921	*P* = 0.671		*P* = 0.683
CRH	*r* = 0.214	*r* = 0.152	*r* = - 0.042	*r* = 1
	*P* = 0.036	*P* = 0.138	*P* = 0.683	

*r*—Spearman correlation coefficient.

*P* < 0.05 is significant.

GAD-7—Generalized Anxiety Disorder 7-item Scale.

PHQ-9—The Patient Health Questionnaire 9-item depression module.

BDNF—Brain-derived neurotrophic factor.

CRH—Corticotropin releasing hormone.

**Figure 1:**
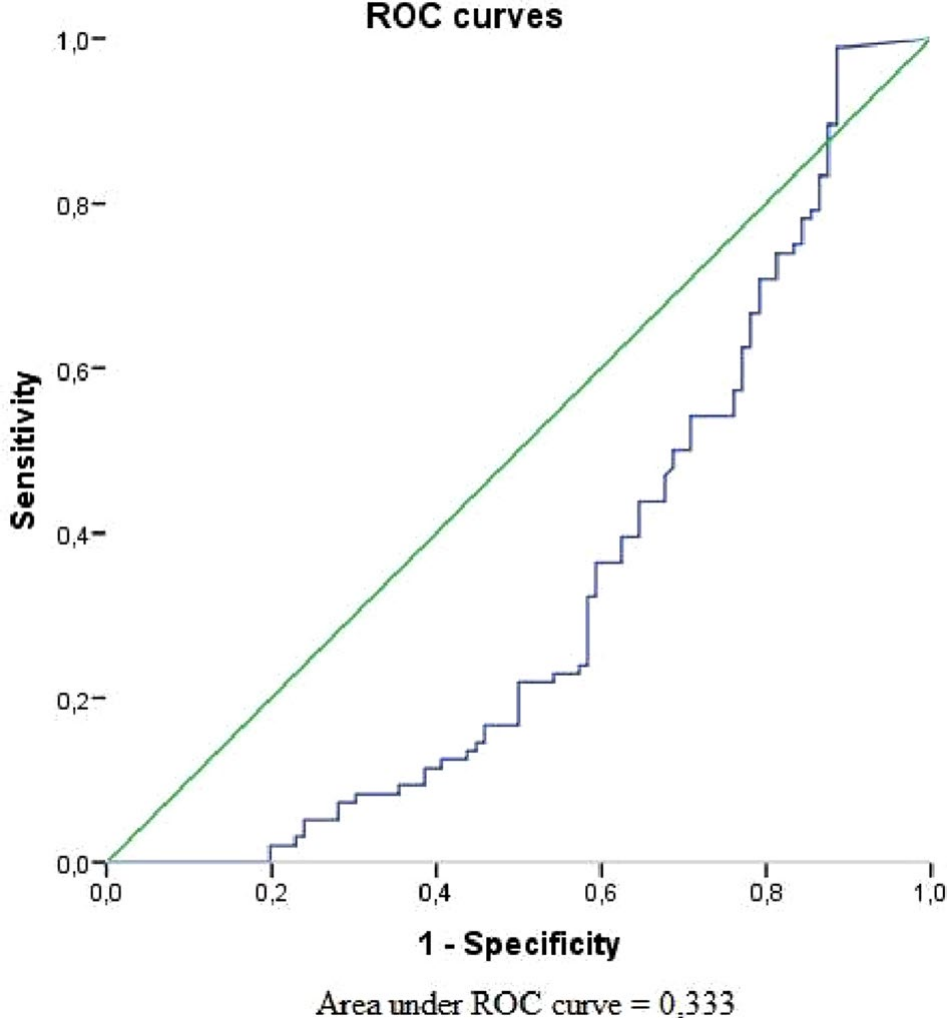
Receiver operating curve graph to evaluate the diagnostic performance of serum BDNF for forecasting of vitiligo event.

**Figure 2:**
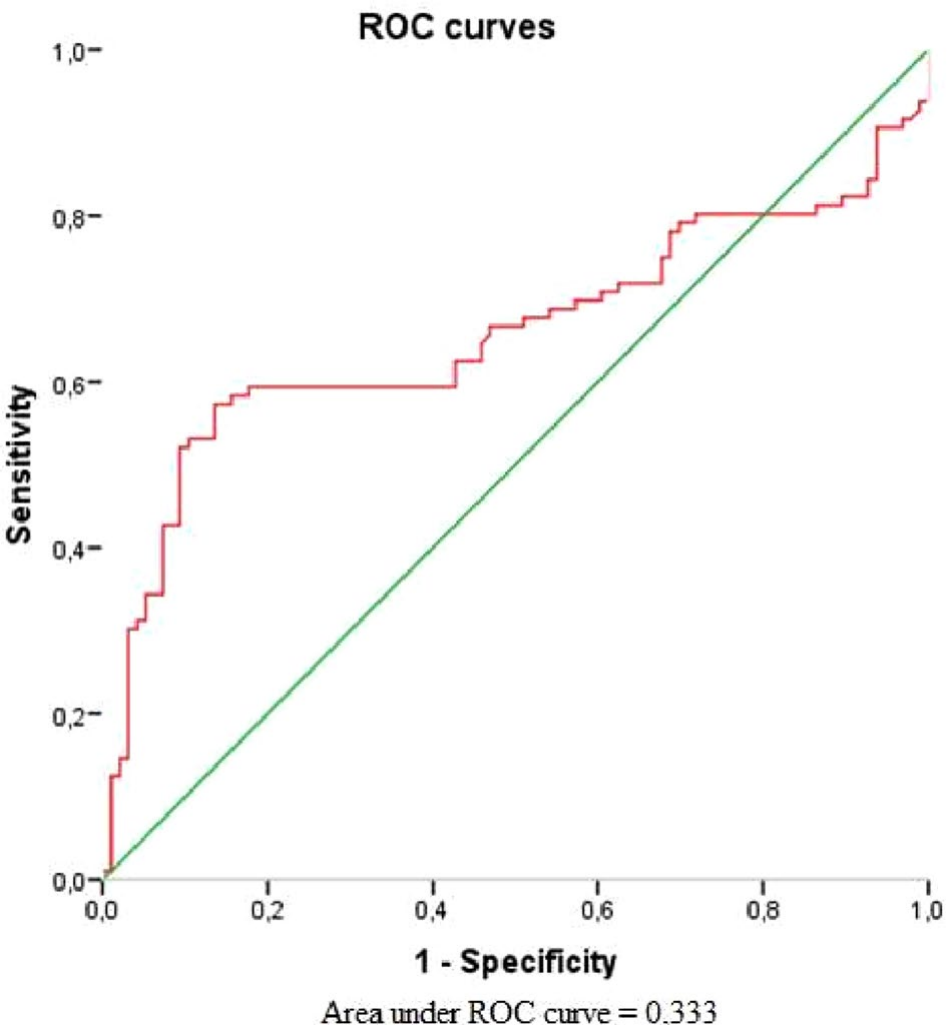
Receiver operating curve graph to evaluate the diagnostic performance of serum CRH for forecasting of vitiligo event.

The area under curve (AUC) for serum BDNF showed no prognostic value of vitiligo (AUC = 0.333) (Fig. 1). Only serum CRH level had poor prognostic value for forecasting of vitiligo event, which was statistically significant (AUC = 0.657) with cut-off point 0.222 at sensitivity equal to 0.938 and specificity equal to 0.990 (Fig. 2).

## Discussion

In the current study, the serum levels of BDNF and CRH with respect to depression and anxiety scores in vitiligo patients and control subjects were compared. The present data demonstrate the significant increase of depression and anxiety in vitiligo patients as compared with healthy controls. The serum levels of BDNF were significantly lower in vitiligo patients than in healthy individuals, while the serum levels of CRH were markedly higher in cases than those in controls. Moreover, we detected significant positive correlations between serum CRH level and GAD-7 score. However, the aforementioned psychometric scales did not correlate significantly with serum BDNF level.

Previous studies revealed the relationship between vitiligo and psychiatric morbidities. Recent meta-analyses reported that vitiligo patients were averagely 5 times more prone to depression as compared with controls^5,6^. Our results agree with these data displaying the higher scores of PHQ-9 scale in patients with vitiligo than in healthy participants. Numerous studies have detected a significant increase of anxiety symptoms among vitiligo patients^9^. In this study, there were significantly lower BDNF levels in vitiligo patients than in controls. In line with these findings, several studies reported the decreased levels of serum BDNF in patients with acne vulgaris and psoriasis in comparison with healthy individuals^24–26^. These results agree with Yanik et al. (2014) who found significant differences in serum BDNF levels between vitiligo and healthy control groups. The authors also attempted to find any correlations between mean serum BDNF level and Beck Depression Inventory and Beck Anxiety Inventory self-reported scales^19^. Our results were consistent with these findings since we also failed to detect associations between serum BDNF levels and PHQ-9 and GAD-7 scores.

However, in our study serum CRH level was significantly higher in vitiligo patients as compared with controls. In addition, serum CRH levels positively correlated with depression and anxiety. Shaker et al. demonstrated significantly higher mean expressions of CRH, and corticotropin releasing hormone receptor 1 (CRHR-1) detected by real-time polymerase chain reaction (PCR) in the depigmented lesions than in control skin. Moreover, significantly higher expressions of CRH and CRH-R1 were correlated with a stress scale^12^. In the current study, there was a positive correlation between serum CRH level and GAD-7. In line with our findings, Tagen et al. reported higher serum levels of CRH in psoriasis patients in contrast with controls. Still, the authors did not find significant correlation between CRH-R1 expression or serum CRH levels, psoriasis area and severity index (PASI) score^27^.

The etiopathogenesis of vitiligo is complex. There are numerous presumed theories of the disease development including oxidative stress hypothesis, genetic predisposition, autoimmune abnormalities, CD8 + T-cells toxicity, biochemical theory, decreased melanocytes regeneration, and neural hypothesis^28–30^. The last theory might play a significant role in interpretation of our main findings. Such, according to neurohumoral theory, the increased levels of certain neuromediators and neuropeptides that are released by neural endings, may launch inflammatory and destructive processes in the skin. This assumption might explain the mechanisms of pathogenesis of segmental vitiligo and its distribution patterns^31^. Numerous studies examined the potential role of BDNF in the development of neuropsychiatric disorders.

Molecular mechanisms of neuronal adaptive plasticity in the brain include interactions between BDNF and tropomysin-related kinase B (TrkB) that, in turn, involves downstream signaling through phosphatidyl inositol-3 kinase (PI3K)-Akt (serine threonine kinase or protein kinase B), Ras/microtubule-associated protein kinase (MAPK), and the phospholipase Cg (PLCg)-Ca^2+^ pathways. According to both in-vitro and clinical studies, the aforementioned pathways, for their part, display the decreased levels of BDNF in situations of stress and depression^32^. Thus, neurotrophins act as inflammatory cytokines, giving signals of activation and survival to effector cells in allergic reactions, and chronic inflammatory skin disorders. Numerous studies reported the crucial role of BDNF in some specific neurobiological processes which may lead to depressive disorders, anxiety-like behavior, and other stress-related mental disorders^33–35^.

Being the part of the proopiomelanocortin system, CRH coordinates pigmentation and immune response in the skin. Stress triggers the release of hormones, especially CRH, from the paraventricular nuclei of the hypothalamus. CRH stimulates the release of ACTH from the anterior pituitary gland. ACTH, in turn, regulates the secretion of glucocorticoids by the adrenal cortex. Cortisol has a suppressive effect on the hypothalamus and the anterior pituitary gland and stimulates the production of epinephrine and norepinephrine by the adrenal glands. In small concentrations, cortisol, adrenaline, and norepinephrine can enhance the immune response in the skin, while in large concentrations they are capable to suppress it^14,36^.

Our paper has some limitations and strengths. Firstly, a cross-sectional design was applied and thus, it was impossible to determine a cause-and-effect relationship between the study variables. Secondly, the self-report measurements for depression and anxiety were used, whereas the gold standard for a psychiatric diagnosis is a structured/semi-structured clinical interview. Thirdly, despite the fact that PHQ-9 and GAD-7 are often used in surveys studying the prevalence of mental disorders, the above-mentioned questionnaires are not intended for people with skin diseases. Thus, it would be desirable to develop and approve a more specific scale for assessing mental health in patients with vitiligo. And finally, we utilized the ELISA kit that detected serum BDNF levels without the identification of precursor and mature forms of this neuropeptide. Nevertheless, our study has several advantages. To the best of our knowledge, this is the first investigation of serum CRH levels in vitiligo patients since other authors performed the CRH measurements in skin only. Also, the current study makes a significant contribution to the limited evidence of relationship between BDNF, vitiligo, and mental comorbidities.

## Data Availability

The data and material are available from the corresponding author on request.

## Abbreviations

BDNF: Brain-derived neurotrophic factor
CRH: Corticotropin releasing hormone
PHQ-9: Patient Health Questionnaire-9
GAD-7: Generalized Anxiety Disorder-7
ELISA: Enzyme-linked immunosorbent assay
ACTH: Adrenocorticotropic hormone
HPA: Hypothalamo-pituitary-adrenal
PPD: Postpartum depression
VES: Vitiligo Extent Score
HRP: Horseradish Peroxidase
TMB: 3,3′,5,5′-Tetramethylbenzidine
OD: Optical density
HS: Higher School
HE: Higher Education
AUR: Area under curve
CRHR-1: Corticotropin releasing hormone receptor 1
MDD: Major depressive disorder
PCR: Polymerase chain reaction
PASI: Psoriasis area and severity index
TrkB: Tropomysin-related kinase B
PI3K: Phosphatidyl inositol-3 kinase
MAPK: Microtubule-associated protein kinase
PLCg: Phospholipase Cg
